# Assessment of causality association between serum adiponectin levels and the risk of Alzheimer’s disease and Parkinson’s disease: a Mendelian randomization study

**DOI:** 10.3389/fneur.2025.1395798

**Published:** 2025-04-30

**Authors:** Jiali Lin, Langhuan Lei, Qiuyu Liang, Xiaozhi Huang, Yanping Ding, Liuxian Pan, Jianrong Yang, Wei Li

**Affiliations:** ^1^Research Center of Health Management, Guangxi Zhuang Autonomous Region People's Hospital, Guangxi Academy of Medical Sciences, Nanning, China; ^2^Department of Health Management, Guangxi Zhuang Autonomous Region People's Hospital, Guangxi Academy of Medical Sciences, Nanning, China

**Keywords:** adiponectin, Alzheimer’s disease, Parkinson’s disease, Mendelian randomization, neurodegenerative diseases

## Abstract

**Background:**

Until recently, the association between circulating adiponectin (ADPN) levels and the risk of Alzheimer’s disease (AD) and Parkinson’s disease (PD) remained unclear.

**Methods:**

We utilized public data from the IEU GWAS database to conduct a two-sample bidirectional Mendelian randomization (MR) analysis and multiple sensitivity analyses. The MR analysis was performed using the aggregated data, with the genetic risk score (GRS) serving as an instrumental variable.

**Results:**

The MR analyses revealed no significant causal association between genetically determined ADPN levels and the risk of AD (OR_IVW_ = 0.852, 95% confidence interval [CI]: 0.586–1.117, *p* = 0.235) or PD (OR_IVW_ = 0.830, 95% CI: 0.780–1.156, *p* = 0.606). Conversely, neither AD nor PD demonstrated any causal association with ADPN levels. The GRS approach yielded similar results (*p* > 0.05). However, it exhibited a negative correlation with interleukin 1β (IL1β, β_IVW_ = −0.31; 95% CI: −0.55 to −0.07, *p* = 0.011). The Cochrane’s Q test and MR-PRESSO analysis revealed no evidence of pleiotropy.

**Conclusion:**

Our findings provide no evidence to substantiate a causal relationship between ADPN levels and the risk of AD and PD or vice versa. However, elevated levels of ADPN may correlate with lower levels of IL1β.

## Introduction

Neurodegenerative diseases (NDs), particularly Alzheimer’s disease (AD) and Parkinson’s disease (PD), are mainly characterized by chronic and selective neuronal cell death, and the onset of these disease is predominantly associated with oxidative stress and neuroinflammation (1). The prevalence of AD and PD increases markedly with aging, especially among the elderly. According to the Alzheimer’s Disease International, approximately 50 million people globally have dementia—a figure projected to triple by 2050 (2). Currently, PD affects 1–2 individuals per 1,000 in the general population and 1% of those over the age of 60 years (3). However, the exact etiology underlying the pathogenic mechanism of these diseases is unclear. Currently, no effective remedies are available for these disorders, and there is a urgent need for new therapeutic targets and biomarkers.

Adiponectin (ADPN), a protein of 244 amino acids located on chromosome 3q27, is a cytokine secreted by adipose tissue. ADPN plays a crucial role in regulating lipid metabolism, energy homeostasis, immune response, inflammation, and insulin sensitivity (4–6). Numerous studies have demonstrated that ADPN is negatively associated with obesity, insulin resistance, type 2 diabetes, atherosclerosis, and cardiovascular disease. These factors can increase the risk of developing AD and PD. ADPN can inhibit the inflammatory response of microglia to AβO while simultaneously promoting the proliferation of hippocampus progenitor cells and Neuro2A cells, thereby performing a neuroprotective function (7). Meanwhile, ADPN exerts an overall anti-inflammatory effect by regulating the expression of cytokines such as IL-6, IFN-r, d TNF-ɑ, IL-1β, and IL-10. Understanding the impact of ADPN on the risk of NDs is imperative (8, 9). However, recent observational studies have demonstrated conflicting associations between ADPN levels and the risk of NDs. Some studies have demonstrated that ADPN levels are comparatively elevated in patients with AD and PD compared to controls, while other studies have revealed significant negative correlations or no associations at all (10–14). Observational studies seek to ascertain whether ADPN exposure causes susceptibility to NDs or if the observed correlation is solely due to other unmeasured confounders. On the other hand, randomized controlled trials present ethical issues that do not substantiate a causal relationship between ADPN and NDs.

**Figure 1:**
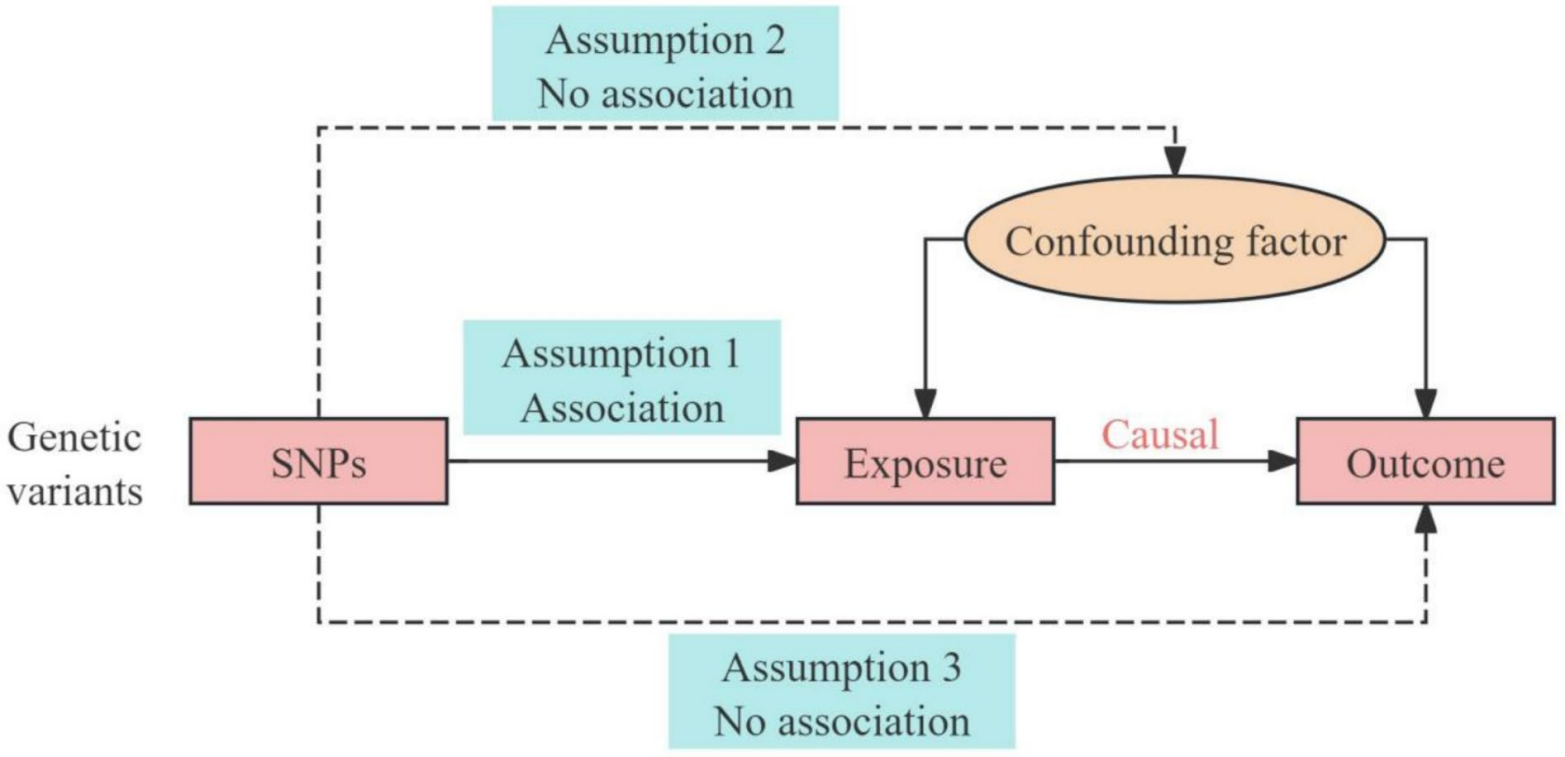
An overview of the study design with three fundamental assumptions of the MR study. The blue boxes represent the three hypotheses of Mendelian randomization, the red boxes represent the causal chain of Mendelian randomization, the solid lines and arrows represent causal effects, and the dashed arrows are not associated with any confounding factors.

Mendelian randomization (MR) offers a new approach to probing the issue of causality in epidemiological research using genetic variants that are robustly associated with exposures as instrumental variables (IVs), enabling more accurate inferences of causality with a particular outcome (15). Genetic variants are randomly combined during gametic formation and are unaffected by environmental and lifestyle factors, thereby minimizing potential confounding factors, measurement errors, or reverse causality inherent in observational studies (16). However, whether MR studies can be used as a reliable tool for inferring causality depends on three core requirements (Figure 1) (17). First, IVs must be strongly associated with the exposure factor. Second, IVs should act independently to confound the exposure–outcome association. Third, IVs must not be correlated with the outcome and only pass through the exposure–outcome pathway (no horizontal pleiotropic effect).

The effect of ADPN on the risk of ND has been inconclusive in observational studies, and the potential causality between them has received limited attention in MR studies. Here, we analyzed the causal relationship between serum ADPN levels and the risk of AD and PD using a two-sample bidirectional MR approach with summarized data from GWAS.

## Methods

### Data sources

GWAS data for ADPN were obtained from the ADIPOGen consortium’s Central European population (*n* = 39,883) (18). The GWAS data for AD and PD were pooled from the International Genomics of Alzheimer’s Project (19) (IGAP, *n* = 17,008 patients with AD and 37,154 controls) and the International Parkinson’s Disease Genomics Consortium (20) (IPDGC, *n* = 33,674 patients and 449,056 controls), respectively. The GWAS data for related traits included body mass index (BMI) [*n* = 322,154, from the Consortium for Genetic Investigation of Human Characteristics (21)] and inflammatory factors [*n* = 3,301, from Sun BB et al. (22)]. These GWAS data were adjusted for demographic characteristics, ensuring the accuracy of genetic information and providing more reliable instrumental variables for MR. All GWAS aggregate data used in this study are publicly available in the IEU GWAS database^1^ and have passed the ethical review process of the IEU database.

### Instrument variable selection

When using GWAS aggregate data to filter IVs for MR studies, a series of specific requirements and parameters must be followed to ensure that the selected instrumental variables are both valid and reliable. First, single nucleotide polymorphisms (SNPs) that show a significant association with exposure factors were screened (*p* < 5 × 10^−8^). Setting thresholds in PLINK (r^2^ > 0.001, maximum distance 10,000 kb) ensures genetic independence between SNPs, reduces redundant information, and avoids strong linkage disequilibrium (LD) bias (23). Subsequently, we extracted the effect estimates for the selected SNPs from each outcome GWAS dataset, ensuring that the SNPs were not directly significantly correlated with the outcome variables. For target SNPs that are missing from the resulting GWAS data, the online tool LDlink^2^ can be used to find proxy SNPs with a high linkage relationship (r^2^ > 0.9) to the target SNPs. In addition, we used the “TwoSampleMR” package to harmonize the exposure and outcome data and to ensure consistency in the analysis variables across different datasets and studies. This process involved removing palindromic SNPs or adjusting the direction of SNP effects, thereby improving the reproducibility and reliability of our findings (Supplementary Tables S1–S3).

**Figure 2:**
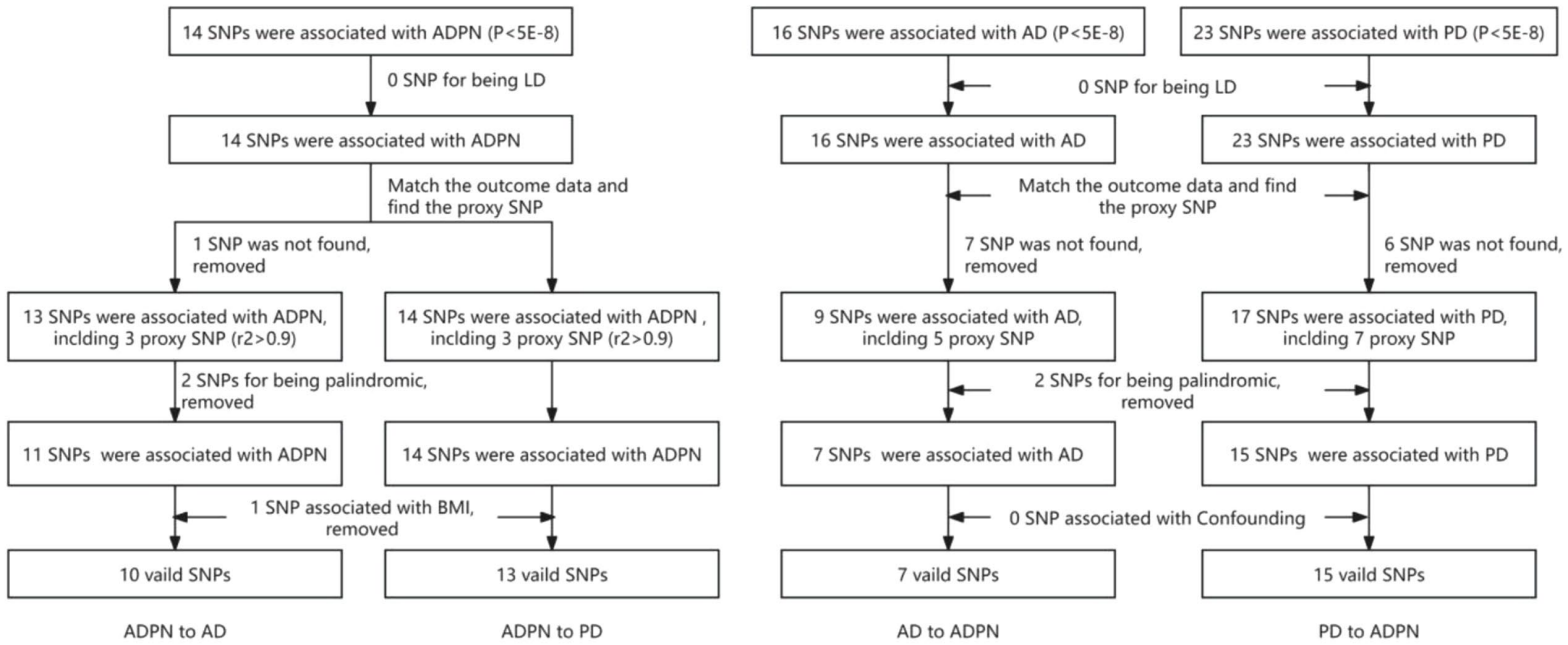
Flowchart of instrumental variable selection. ADPN, adiponectin; AD, Alzheimer’s disease; PD, Parkinson’s disease; SNPs, single-nucleotide polymorphisms; LD, linkage disequilibrium.

We further calculated the F-statistic for each SNP and assessed the variance explained by exposure to reduce potential bias and satisfy the hypothesis that instrumental variables are significantly associated with exposure (Supplementary methods 1). We evaluated the relationship between ADPN and ND-related features, including BMI and inflammatory factors, to test the hypothesis that instrumental variables are not associated with confounders. We adopted various methods (Cochran’s Q test, MR-Egger regression intercept term test, “leave-one-out” sensitivity analysis, and MR-PRESSO analysis) to ensure that the relationship between the instrumental variables and outcome variables was transmitted only through exposure factors, avoiding direct effects and pleiotropy. Using PhenoScannerV2 and GWAS catalog retrieval, potential pleiotropic SNPs were gradually removed (for more details, refer to the section on Heterogeneity and Sensitivity Tests). The IV filtering process is shown in Figure 2.

### Statistical analyses

#### Mendelian randomization analysis

Three MR methods were employed to estimate the causal relationship between exposure and outcomes: (1) The inverse-variance weighted (IVW) method, which is based on the premise that all instrumental variables are valid without evidence of directional pleiotropy and is considered the most reliable MR method. (2) MR-Egger regression, where the slope and intercept represent causality and pleiotropy, respectively, although this method comes at the expense of lowered statistical power. (3) The weighted median method, which is a more robust MR approach and is particularly useful when up to half of the weight comes from invalid IVs. R software (version 4.3.1) and the R software packages “TwosampleMR,” “gtx,” “MR-PRESSO,” and “forestploter” were used for statistical analysis. All causal effect estimates (beta coefficients) were converted to odds ratios (ORs). A *p*-value of < 0.05 was considered statistically significant.

#### Genetic risk score

The core step of the genetic risk score (GRS) method was to generate an individualized, comprehensive genetic risk score by weighting the effects of genetic variants (i.e., risk SNPs) associated with a specific exposure phenotype. The specific method includes the following steps: (1) IV selection. SNPs significantly associated with exposure phenotypes were screened from the previously collected GWAS data. To avoid pleiotropic or confounding effects, SNPs that may be associated with other phenotypes were further eliminated—for example, by evaluating their effects outside the exposed phenotype to exclude pleiotropic loci. At the same time, SNPs with palindromic sequences (such as A/T or C/G), where alleles were not clearly referenced, were either cleaned or deleted to ensure the reliability of the analysis. (2) Extraction and weighting of the SNP effect. Using the “gtx” package in R and its grs.summary module, the exposed SNP effect values (usually expressed as *β*) were extracted from the GWAS summary data. The effect value of each SNP was weighted, with the weighting coefficient determined according to the strength of the SNP’s association with the exposure phenotype (i.e., the absolute value of the beta value). The personalized weighted GRS was calculated using the following formula: ${GRS}_{i}=\overset{n}{\underset{j=1}{\sum }}\beta_{j}G_{ij}$ , where ${GRS}_{i}$ is the genetic risk score of individual i, $\beta_{j}$ is the effect value of j SNP, and $G_{ij}$ is the genotype of individual i at j SNP. 0, 1, and 2 indicate the copy number of the alleles. (3) Correlation analysis between the GRS and outcome phenotype. In the statistical analysis, the GRS was treated as a continuous variable and the relationship between the GRS and outcome phenotype was assessed using linear regression models (for continuous outcome variables) or logistic regression models (for binary outcome variables). (4) Heterogeneity test and bias assessment. To verify the consistency of the contributions of the selected SNPs to the GRS, Cochran’s Q test was used to assess the effect of heterogeneity among SNPs.

#### Heterogeneity and pleiotropy tests

We performed heterogeneity and sensitivity tests to further evaluate the validity of the results. Heterogeneity among the SNPs was assessed using Cochran’s Q test, and a *p*-value of < 0.05 was considered statistically significant (suggesting the possibility of heterogeneity). The presence of directional horizontal pleiotropy was assessed according to the intercept term from MR-Egger regression (*p* < 0.05 indicated the possibility of pleiotropy). We performed a “leave-one-out” sensitivity analysis for each SNP to investigate the potential impact of the SNPs on causal estimation. MR-PRESSO analysis was performed to identify outliers to adjust the estimates, thereby improving the reliability of the IVs (*p* < 0.05 indicated a significant global). The PhenoScannerV2 database^3^ and GWAS catalog^4^ were searched for potential confounding traits, and possible pleiotropic instrumental variables were gradually removed (Supplementary Tables S4–S6).

#### Sample size and power calculations

We referred to the method of calculating power.^5^ The results revealed that both the bidirectional MR analysis from exposure to outcome and the reverse MR analysis from outcome to exposure had sufficient power to detect statistically significant effects and met the necessary sample size requirements (Supplementary Table S7). This supports the conclusion that the observed associations are unlikely to have occurred by chance.

## Results

### MR estimates of ADPN and AD

Table 1 and Supplementary Figure S3 show that no significant association was observed between ADPN and the risk of AD using any of the three MR methods (IVW: OR_IVW_ = 0.852, 95% CI: 0.586–1.117, *p* = 0.235; OR_WM_ = 0.808, 95% CI: 0.539–1.078, *p* = 0.122; OR_MR-Egger_ = 0.740, 95% CI: 0.312–1.168, *p* = 0.205). The Cochran’s Q statistics indicated almost no heterogeneity between the IVs (Q_IVW_ = 15.754, *p* = 0.072; Q_MR-Egger_ = 14.502, *p* = 0.070), and the MR-Egger intercept test showed no evidence of horizontal pleiotropy (intercept = 0.011; *p* = 0.430). No potential outliers were found in the MR-PRESSO (global *p* = 0.117) (Table 2). The “Leave-one-out analysis” revealed that no SNP significantly contributed to estimate the causal association between ADPN levels and the risk of AD (Supplementary Figure S1A). These tests supported the reliability of the MR results.

**Table 1:** MR results for the relationships between ADPN and NDs.

Exposure-outcome	Number of SNPs	Method	OR (95%CI)	*p*-value
ADPN-AD	10^a^	IVW	0.852 (0.586–1.117)	0.235
	10^a^	Weighted median	0.808 (0.539–1.078)	0.122
	10^a^	MR Egger	0.740 (0.312–1.168)	0.205
ADPN-PD	13^b^	IVW	0.830 (0.780–1.156)	0.606
	13^b^	Weighted median	0.823 (0.649–1.045)	0.113
	13^b^	MR Egger	0.756 (0.493–1.020)	0.060
AD-ADPN	7^c^	IVW	0.981 (0.946–1.018)	0.318
	7^c^	Weighted median	0.993 (0.950–1.038)	0.743
	7^c^	MR Egger	1.023 (0.890–1.177)	0.759
PD-ADPN	15^d^	IVW	1.003 (0.982–1.024)	0.795
	15^d^	Weighted median	1.002 (0.976–1.029)	0.884
	15^d^	MR Egger	1.029 (0.973–1.088)	0.330

a Rs12051272 was not matched in the AD GWAS summary data, rs601339 had potential multieffect effects, and palindromic SNPs (rs2980879 and rs7964945) were removed.

b Rs601339 had potential multieffect effects and was deleted.

c Seven SNPs were not matched in the ADPN GWAS summary data, and palindromic SNPs (rs12972156 and rs12977604) were removed.

d Six SNPs were not matched in the ADPN GWAS summary data, and palindromic SNPs (rs10451230 and rs35265698) were removed.

**Table 2:** The heterogeneity and sensitivity results for the association between ADPN and the risk of AD and PD.

Exposure-outcome	MR-PRESSO	Cochran’s heterogeneity statistic				Horizontal pleiotropy		
	Global *p-*value	IVW		MR Egger		MR-Egger		
		Q	*p*	Q	*p*-value	Intercept	SE	*p*-value
ADPN-AD	0.117	15.754	0.072	14.502	0.070	0.011	0.013	0.430
ADPN-PD	0.561	10.984	0.530	7.864	0.725	0.017	0.009	0.105
AD-ADPN	0.409	8.347	0.214	7.761	0.170	−0.006	0.010	0.566
PD-ADPN	0.182	17.806	0.216	16.564	0.220	−0.004	0.004	0.341

ADPN, adiponectin; AD, Alzheimer’s disease; PD, Parkinson’s disease; IVW, inverse-variance weighted.

In contrast, no evidence of a causal association was observed between genetic predisposition to AD and ADPN (*p* > 0.05) (Table 1). Similarly, no significant horizontal pleiotropy was observed (intercept = −0.006; *p* = 0.566), nor was there any evidence of heterogeneity (Q_IVW_ = 8.347, *p* = 0.214; Q_MR-Egger_ = 7.761, *p* = 0.170) or potential outliers (global *p* = 0.409) (Table 2; Supplementary Figure S1B).

### MR estimates of ADPN and PD

The IVW method did not find any statistically significant association between genetically determined ADPN and PD (OR = 0.830, 95% CI: 0.780–1.156, *p* = 0.606). In addition, the MR-Egger regression (OR: 0.756, 95% CI: 0.493–1.020, *p* = 0.060) and weighted median methods (OR: 0.823, 95% CI: 0.649–1.045, *p* = 0.113) yielded similar results, with slightly wider confidence intervals (Table 1; Supplementary Figure S4). The Cochran’s Q test did not detect significant heterogeneity between the estimates of the IVs. The “leave-one-out” analysis revealed that no single SNP influenced the IVW causal association estimates (Table 2; Supplementary Figure S2A).

Similarly, our results did not reveal a significant causal relationship between the risk of PD and ADPN levels, with all three methods yielding consistent estimates in the same direction (OR_IVW_: 1.003, 95% CI_IVW:_ 0.982–1.024, *P*_IVW_ = 0.795; OR_WM_: 1.002, 95% CI_WM_: 0.975–1.029, *P*_WM_ = 0.887; OR_MR-Egger_: 1.029, 95% CI_MR-Egger_: 0.973–1.085, *P*_MR-Egger_ = 0.330) (Table 1). Furthermore, no potential outliers (global *p* = 0.182) were identified, and heterogeneity (Q_IVW_ = 17.806, *P*_IVW_ = 0.216; Q_MR-Egger_ = 16.564, *P*_MR-Egger_ = 0.220) and horizontal pleiotropy (intercept = −0.004, *p* = 0.341) were not observed in the causal relationship between PD and ADPN (Table 2; Supplementary Figure S2B).

### GRS estimates of ADPN and the risk of NDs

As for the GRS exposure and outcome, no causal relationship was observed between serum ADPN and the risk of AD (OR = 0.851, 95% CI: 0.661–1.041, *p* = 0.100 in the forward direction; OR = 1.006, 95% CI: 0.994–1.017, *p* = 0.345 in the reverse direction) (Table 3). Consistently, the GRS results showed no causal relationship between ADPN and the risk of PD. In addition, the results of the study revealed that no heterogeneous effect on the risk of the outcome was observed in the direction in relation to the estimated effect of exposure on the risk of the outcome (All *P*-Het > 0.05).

**Table 3:** The effects of GRS_ADPN_ on NDs and GRS_NDs_ on ADPN.

Exposure	Outcome	OR (95%CI)	*p*-value	Qrs	*P*-Het
ADPN	AD	0.851 (0.661–1.041)	0.100	19.925	0.069
ADPN	PD	0.949 (0.775–1.124)	0.561	16.525	0.222
PD	ADPN	0.999 (0.981–1.018)	0.931	21.112	0.174
AD	ADPN	1.006 (0.994–1.017)	0.345	11.025	0.200

ADPN, adiponectin; AD, Alzheimer’s disease; PD, Parkinson’s disease; OR, odds ratio.

### MR estimates of ADPN and ND-related traits

**Figure 3:**
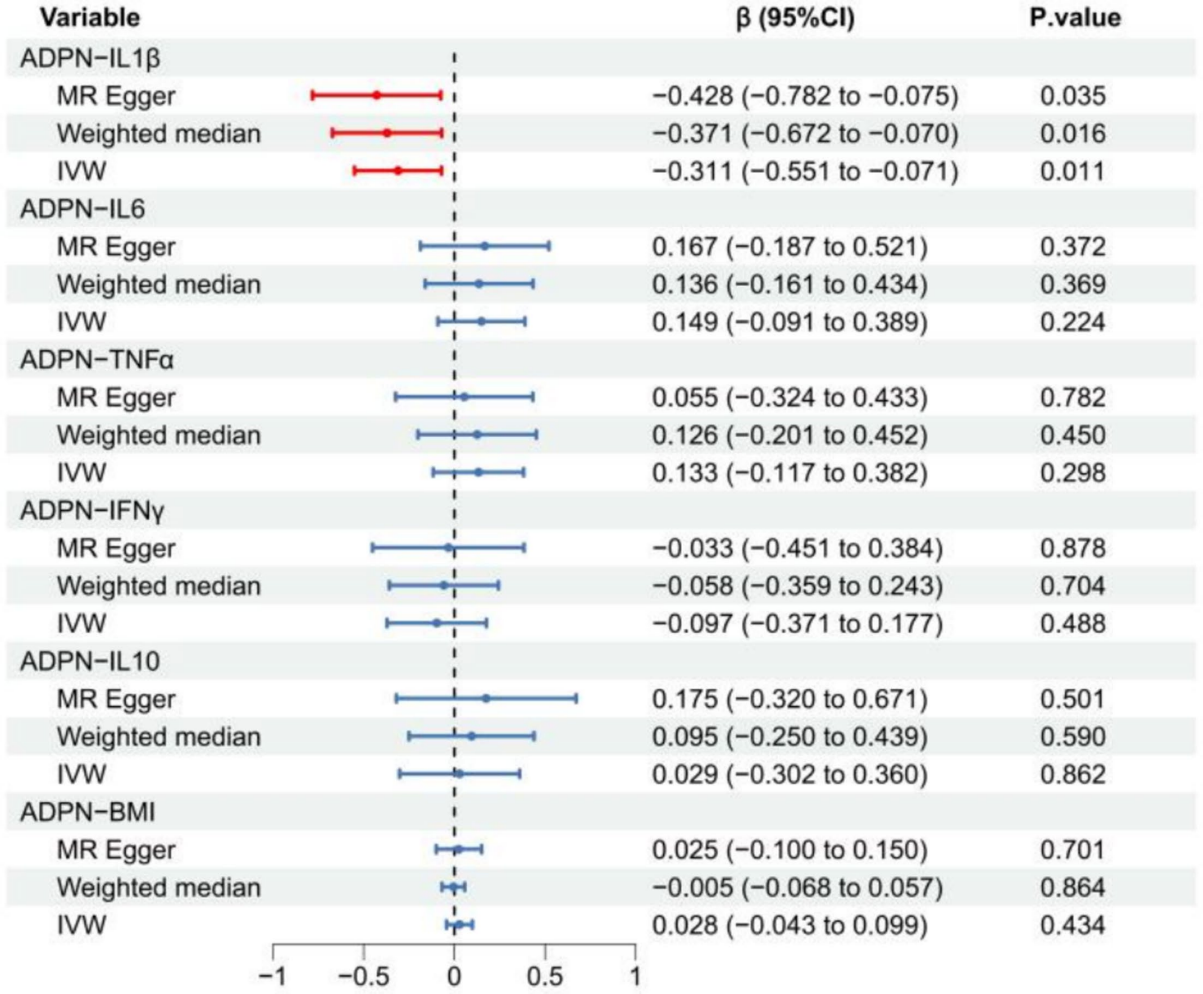
MR estimate plot showing the effect of ADPN on ND-related traits. The red line indicates statistical significance (*p* < 0.05), while the blue line indicates no statistical significance (*p* > 0.05). ADPN, adiponectin; IVW, inverse-variance weighted.

The MR results revealed a negative correlation between ADPN and IL1β (β_IVW_ = −0.31; 95% CI: −0.55 to −0.01, *p* = 0.011; β_Weighted median_ = −0.37; 95% CI: −0.67 to −0.07, *p* = 0.016; β_MR-Egger_ = −0.43; 95% CI: −0.78 to −0.07, *p* = 0.035). However, the forest plot showed that none of the three MR methods demonstrated a causal association between ADPN and any other ND-related traits (*p* > 0.05) (Figure 3; Supplementary Table S8).

## Discussion

This extensive two-way MR study found no evidence supporting a causal relationship between ADPN levels and susceptibility to AD and PD, and vice versa. These results are robust because multiple heterogeneity and sensitivity analyses were performed to detect and eliminate any potential genetic pleiotropy.

ADPN has anti-inflammatory, anti-atherosclerotic, and insulin-sensitizing properties and is highly heritable, with an estimated genetic risk of 0.40–0.88 based on twin and sibling studies (24, 25). Growing neurobiological evidence indicates that ADPN exerts protective effects in various regions of the central nervous system by binding to AdipoR1 and AdipoR2. However, in a 13-year cohort study from the Framingham Heart Study, ADPN was not associated with all-cause dementia (HR = 1.14, 95% CI: 0.97–1.34) or AD (HR = 1.19, 95% CI: 0.98–1.43). Further subgroup analysis revealed that female sex and higher baseline ADPN levels were significantly associated with an increased risk of all-cause dementia and AD (HR = 1.13, 95% CI: 1.07–1.61; HR = 1.32, 95% CI: 1.06–1.65), while no significant associations were observed in male sex (*p* > 0.05). However, no association was found after adjusting for age, BMI, weight change, APOEε4 allele, and other confounding factors (11). Consistent with the findings of cohort studies (26–28) across most populations, our MR study also did not find a significant causal relationship between genetically determined ADPN levels and AD. In the above-mentioned studies, we found that the differences between observational studies may be attributed to the fact that confounders, including age, obesity, and metabolic diseases, were not completely excluded, all of which are associated with chronic inflammation. However, our MR study selected SNPs significantly associated with ADPN from the GWAS dataset and removed ADPN SNPs associated with age, obesity, inflammation, and APOE sites to minimize type I errors. Therefore, the results of MR studies may be more reliable.

This phenomenon is reflected in studies examining ADPN and PD. The results of our MR study confirm the results of previous observational studies (29, 30), indicating no association between serum ADPN and the risk of PD, although some studies have indicated a positive correlation. For instance, Hiroshi et al. (31) reported that compared to the control group (13.3 ± 7.0 mg/L), ADPN levels in patients with PD (18.6 ± 8.3 mg/L), patients with MSA-P (16.5 ± 9.3 mg/L), and patients with PSP (13.8 ± 6.7 mg/L) were significantly increased (*p* < 0.05). ADPN levels in patients with PD were significantly higher than those in age-matched morbidly obese individuals. However, they were similar to those in normal-weight, healthy young individuals. In non-obese individuals, men (7.7 ± 3.1 μg/mL) had lower mean ADPN levels than women (10.6 ± 7.3 μg/mL). In addition, ADPN was positively correlated with HDL-c concentration in patients with PD, indicating that ADPN has a protective effect on cardiovascular events, including anti-inflammation and anti-atherosclerosis properties in patients with PD (32). According to the above-mentioned studies, sex and obesity are confounding factors affecting the level of ADPN. The discrepancy between our results and those of observational epidemiologic studies could be attributed to small sample sizes, lack of adjustment for potential confounders, and reverse causality in the previous studies.

In our MR study, which utilized the IVW, weighted median, and MR-Egger methods, we consistently observed a negative correlation between ADPN and IL1β, with no evidence of heterogeneity or pleiotropy, following the exclusion of SNPs with high genetic overlap or those associated with IL1β. This suggests that adiponectin may play a role in regulating neuroinflammation. Low levels of ADPN may indicate a metabolic disorder (obesity and diabetes), which, in itself, may promote systemic low-grade inflammation, further exacerbating the inflammatory response in the nervous system (7, 33). Low levels of adiponectin (ADPN) enhance immune cell reactivity by inhibiting the AMPK and PPAR-*γ* signaling pathways; promote the release of pro-inflammatory factors such as TNF-*α*, IL-6, and IL-1; disrupt the integrity of the blood–brain barrier; activate microglia and astrocytes; and exacerbate neuroinflammation and neurodegeneration. On the contrary, adiponectin can positively regulate hippocampal synaptic plasticity by activating the AdipoR1/AMPK signaling pathway and improve spatial learning ability and working memory function in mice (34, 35). Neurogenic inflammation can harm nerve cells and synaptic connections, increase levels of Aβ and tau, and induce neurodegeneration through multiple pathways, including ROS, RNS, GMF, and PAP-2, resulting in nerve inflammation and a vicious cycle of pathology (36). However, our study did not demonstrate a causal relationship between ADPN and NDs, which contrasts with some observational studies. As mentioned earlier, lifestyle or sociodemographic factors may not obscure the genetic predictors of exposure. Utilizing genetic tools to examine the association between ADPN and NDs, our MR analysis revealed that previous observational associations may not reflect a true causal relationship. The potential role of ADPN in NDs may be indirect, mediated by its effect on systemic inflammation, particularly through IL1β, rather than directly influencing the disease. Although our MR findings do not indicate a direct causal relationship between ADPN and NDs, the negative association between ADPN and IL1β provides valuable insights into the inflammatory mechanisms implicated in neurodegeneration.

This study systematically investigated the potential causal relationship between ADPN levels and the risk of NDs (AD and PD), using both bidirectional MR analysis and GRS methods. Despite its strengths, this MR study has some limitations that should be taken into account. First, the MR analysis relies on three core assumptions. However, these assumptions may not be fully valid in actual studies, especially the third assumption, which could be influenced by horizontal pleiotropy. To address this, we conducted rigorous screening of candidate SNPs and carried out multiple sensitivity analyses (including MR-Egger regression, the weighted median method, and others) and heterogeneity tests to minimize potential bias and verify the robustness of the results. The results showed that the MR estimates were stable and no significant pleiotropy was found, but we could not completely rule out the possibility of residual bias. Second, the data for this study were mainly based on aggregated GWAS results from the European population, so the applicability of the study’s conclusions to other ethnic groups remains unclear. Considering that racial differences may influence the relationship between genetic structure and disease risk, follow-up studies are necessary to verify the results in multi-ethnic samples, which would improve the generalizability and broad applicability of the results. Third, because the GWAS data we used were publicly available aggregated data, detailed demographic and clinical characteristics (such as age of onset and disease subtypes) at the individual level were not accessible, therefore further detailed subgroup analysis was not possible. This, to some extent, limits our exploration of the potential effects of ADPN in different subpopulations.

## Conclusion

These bidirectional MR studies did not demonstrate a causal relationship between genetically determined ADPN levels and susceptibility to AD and PD, or vice versa. However, ADPN levels were negatively correlated with IL1β. Further investigation is required to understand the mechanism and to gather more epidemiological and genetic data to test this hypothesis.

## Data Availability

The original contributions presented in the study are included in the article/Supplementary material, further inquiries can be directed to the corresponding authors.

## Footnotes

^1^ https://gwas.mrcieu.ac.uk/

^2^ https://ldlink.nci.nih.gov/

^3^ http://www.phenoscanner.medschl.cam.ac.uk/

^4^ https://www.ebi.ac.uk/gwas/summary-statistics

^5^ https://shiny.cnsgenomics.com/mRnd/
