# Supplementary material for: Assessment of causality association between serum adiponectin levels and the risk of Alzheimer’s disease and Parkinson’s disease: a Mendelian randomization study
Source: Front Neurol. 2025 Apr 30;16:1395798. doi: 10.3389/fneur.2025.1395798 (PMC12075267; doi:10.3389/fneur.2025.1395798)
Supplement: Supplementary file 1 [file Data_Sheet_1.docx]

Supplementary Materials

1. Table S1: Genome-wide significant SNPs (n = 14) for adiponectin (*P* < 5×10^-8^).
2. Table S2: Genome-wide significant SNPs (n = 16) for AD (*P* < 5×10^-8^).
3. Table S3: Genome-wide significant SNPs (n = 23) for PD (*P* < 5×10^-8^).
4. Table S4: GWAS linked traits of 14 instrument SNPs of adiponectin.
5. Table S5: GWAS linked traits of 7 instrument SNPs of AD.
6. Table S6: GWAS linked traits of 15 instrument SNPs of PD.
7. Table S7: The sample size and power calculations for MR analyses.
8. Table S8: The heterogeneity and sensitivity results of ADPN and NDs relevant traits.
9. Fig. S1: Leave-one-out plot to assess if a single SNP drives association between adiponectin and AD.
10. Fig. S2: Leave-one-out plot to assess if a single SNP drives association between adiponectin and PD.
11. Fig. S3: Scatter plots of causal associations between adiponectin with AD and PD.
12. Fig. S4: Scatter plots of causal associations between ADPN and PD
13. [Supplementary methods](javascript:;)

Table S1. Genome-wide significant SNPs (n = 14) for ADPN (*P* < 5×10^-8^).

| SNP | Proxy SNP^b^ | Effect allele | other allele | Exposure for ADPN | |  | Outcome | | | | | Variance  explained (R^2^) | F statistic |
| --- | --- | --- | --- | --- | --- | --- | --- | --- | --- | --- | --- | --- | --- |
|  |  |  |  |  |  |  | AD | |  | PD | |  |  |
|  |  |  |  | Beta | SE |  | Beta | SE |  | Beta | SE |  |  |
| rs2062632 |  | C | T | -0.055 | 0.006 |  | -0.003 | 0.020 |  | -0.003 | 0.022 | 0.0030 | 86.052 |
| rs17366568 |  | A | G | -0.154 | 0.009 |  | 0.059 | 0.034 |  | 0.030 | 0.026 | 0.0125 | 314.839 |
| rs1108842 | rs11130324 | C | A | 0.030 | 0.004 |  | -0.008 | 140.015 |  | 0.039 | 0.017 | 0.0016 | 46.642 |
| rs1597466 | rs7617025 | T | G | -0.044 | 0.008 |  | -0.066 | 0.025 |  | 0.007 | 0.029 | 0.0011 | 33.673 |
| rs6810075 |  | C | T | -0.066 | 0.005 |  | 0.034 | 0.017 |  | -0.012 | 0.018 | 0.0066 | 192.333 |
| rs7615090 |  | G | T | -0.058 | 0.008 |  | 0.010 | 0.027 |  | 0.015 | 0.029 | 0.0022 | 47.199 |
| rs2980879a |  | T | A | 0.030 | 0.005 |  | 0.003 | 0.016 |  | 0.018 | 0.018 | 0.0014 | 34.817 |
| rs7955516 |  | C | A | 0.026 | 0.005 |  | 0.028 | 0.017 |  | 0.001 | 0.019 | 0.0011 | 33.144 |
| rs601339 |  | G | A | 0.039 | 0.006 |  | -0.036 | 0.020 |  | 0.052 | 0.023 | 0.0016 | 46.521 |
| rs7964945a |  | A | T | 0.037 | 0.006 |  | 0.025 | 0.024 |  | 0.016 | 0.025 | 0.0011 | 33.001 |
| rs8042532 | rs8039455 | G | T | -0.340 | 0.055 |  | 0.040 | 0.061 |  | 0.079 | 0.057 | 0.0048 | 37.578 |
| rs12051272 |  | T | G | -0.277 | 0.018 |  | - | - |  | 0.015 | 0.093 | 0.0147 | 233.266 |
| rs2927324 |  | T | C | 0.032 | 0.005 |  | -0.010 | 0.016 |  | 0.019 | 0.022 | 0.0017 | 48.826 |
| rs731839 |  | A | G | 0.037 | 0.005 |  | -0.010 | 0.017 |  | 0.012 | 0.019 | 0.0020 | 57.312 |

Abbreviations: ADPN, adiponectin; AD, Alzheimer’s disease; PD, Parkinson's disease; SNP, single-nucleotide polymorphism; Beta, The effect size; SE,

The standard error of the effect size.

^a^Two SNPs (rs2980879, rs7964945) of palindrome between ADPN and AD were screened out.

^b^Proxy SNP reported where the targeted SNP was not available in the outcome datasets, and the effect allele and beta (SE) reported for proxy SNP.

Table S2. Genome-wide significant SNPs (n = 16) for AD (*P* < 5×10^-8^).

| SNP | Proxy SNPb | Effect allele | other allele | Exposure for AD | |  | Outcome for ADPN | | Variance  explained (R^2^) | F statistic |
| --- | --- | --- | --- | --- | --- | --- | --- | --- | --- | --- |
|  |  |  |  | Beta | SE |  | Beta | SE |  |  |
| rs1752684 | rs2296160 | G | A | -0.154 | 0.020 |  | -0.001 | 0.005 | 0.0060 | 61.815 |
| rs4663105 |  | C | A | 0.184 | 0.017 |  | - | - | 0.0163 | 114.067 |
| rs9381563 |  | T | C | -0.097 | 0.017 |  | 0.004 | 0.005 | 0.0041 | 34.004 |
| rs9272561 |  | A | G | -0.136 | 0.023 |  | - | - | 0.0091 | 34.964 |
| rs10808026 | rs11767557 | A | C | -0.139 | 0.021 |  | -0.004 | 0.006 | 0.0058 | 45.726 |
| rs7982 | rs11136000 | G | A | 0.140 | 0.017 |  | -0.002 | 0.005 | 0.0097 | 71.993 |
| rs10792832 |  | G | A | 0.130 | 0.016 |  | -0.011 | 0.005 | 0.0079 | 64.898 |
| rs11218343 |  | C | T | -0.270 | 0.041 |  | -0.013 | 0.014 | 0.0060 | 43.271 |
| rs72924659 |  | T | C | -0.141 | 0.020 |  | - | - | 0.0083 | 51.972 |
| rs12590654 |  | A | G | -0.097 | 0.018 |  | - | - | 0.0042 | 30.063 |
| rs8093731 |  | T | C | -0.614 | 0.112 |  | 0.052 | 0.043 | 0.0089 | 29.855 |
| rs12972156 | rs2075650 | G | C | 1.140 | 0.026 |  | 0.011 | 0.007 | 0.0353 | 1982.684 |
| rs11881756 |  | C | T | -0.183 | 0.029 |  | - | - | 0.0007 | 40.272 |
| rs117310449 |  | T | C | 1.214 | 0.097 |  | - | - | 0.0029 | 157.807 |
| rs4147929 |  | G | A | -0.135 | 0.022 |  | - | - | 0.0055 | 36.215 |
| rs12977604 | rs5157 | G | C | 0.151 | 0.018 |  | 0.001 | 0.006 | 0.0012 | 67.080 |

Abbreviations: ADPN, adiponectin; AD, Alzheimer’s disease; PD, Parkinson's disease; SNP, single-nucleotide polymorphism; Beta, The effect size;

SE, The standard error of the effect size.

^a^Two SNPs (rs12972156, rs12977604) of palindrome between AD and ADPN were screened out.

^b^Proxy SNP reported where the targeted SNP was not available in the outcome datasets, and the effect allele and beta (SE) reported for proxy SNP.

Table S3. Genome-wide significant SNPs (n = 23) for PD (*P* < 5×10^-8^).

| SNP | Proxy SNPb | Effect allele | other allele | Exposure for PD | |  | Outcome for ADPN | | Variance  explained (R^2^) | F statistic |
| --- | --- | --- | --- | --- | --- | --- | --- | --- | --- | --- |
|  |  |  |  | Beta | SE |  | Beta | SE |  |  |
| rs35749011 | rs2230288 | A | G | 0.7508 | 0.0659 |  | -0.0002 | 0.020 | 0.0003 | 129.801 |
| rs823106 |  | C | G | -0.1492 | 0.0239 |  | 0.014 | 0.006 | 0.0001 | 38.971 |
| rs4613239 |  | G | C | 0.1784 | 0.0248 |  | - | - | 0.0001 | 51.747 |
| rs6741007 | rs7599054 | G | T | -0.1233 | 0.0175 |  | 0.002 | 0.004 | 0.0001 | 49.642 |
| rs4488803 |  | A | G | -0.1136 | 0.0199 |  | 0.018 | 0.010 | 0.0001 | 32.587 |
| rs10513789 |  | G | T | -0.1596 | 0.0219 |  | -0.008 | 0.005 | 0.0001 | 53.110 |
| rs7695720 |  | C | A | -0.1255 | 0.0208 |  | -0.002 | 0.005 | 0.0001 | 36.405 |
| rs34311866 |  | C | T | 0.2272 | 0.0231 |  | - | - | 0.0002 | 96.737 |
| rs4698412 |  | A | G | 0.1258 | 0.0168 |  | 0.006 | 0.005 | 0.0001 | 56.072 |
| rs356203 |  | T | C | -0.2398 | 0.0178 |  | - | - | 0.0004 | 181.492 |
| rs75646569 | rs7718028 | G | T | 0.1916 | 0.0266 |  | 0.011 | 0.007 | 0.0001 | 51.883 |
| rs35265698 |  | G | C | -0.2 | 0.0303 |  | 0.032 | 0.038 | 0.0001 | 43.569 |
| rs858295 | rs1637192 | G | A | -0.1039 | 0.0176 |  | 0.003 | 0.004 | 0.0001 | 34.850 |
| rs620490 |  | G | T | -0.1174 | 0.019 |  | -0.001 | 0.005 | 0.0001 | 38.179 |
| rs144814361 |  | T | C | 0.4411 | 0.068 |  | - | - | 0.0001 | 42.078 |
| rs329647 |  | C | G | -0.1133 | 0.0178 |  | 0.000 | 0.005 | 0.0001 | 40.515 |
| rs75505347 |  | T | C | 0.3917 | 0.0674 |  | - | - | 0.0001 | 33.774 |
| rs10847864 |  | T | G | 0.1274 | 0.0179 |  | - | - | 0.0001 | 50.656 |
| rs4774417 | rs2414739 | A | G | 0.1052 | 0.0192 |  | -0.005 | 0.005 | 0.0001 | 30.021 |
| rs12934900 | rs1458202 | T | A | 0.1215 | 0.0184 |  | -0.004 | 0.005 | 0.0001 | 43.603 |
| rs58879558 | rs1052594 | C | T | -0.2383 | 0.025 |  | -0.005 | 0.006 | 0.0002 | 90.859 |
| rs10451230 | rs1989856 | T | A | -0.096 | 0.0175 |  | 0.007 | 0.004 | 0.0001 | 30.093 |
| rs4588066 |  | A | G | 0.1046 | 0.0178 |  | -0.001 | 0.005 | 0.0001 | 34.532 |

Abbreviations: ADPN, adiponectin; AD, Alzheimer’s disease; PD, Parkinson's disease; SNP, single-nucleotide polymorphism; Beta, The effect size;

SE, The standard error of the effect size.

^a^Two SNPs (rs10451230, rs35265698) of palindrome between ADPN and PD were screened out

^b^Proxy SNP reported where the targeted SNP was not available in the outcome datasets, and the effect allele and beta (SE) reported for proxy SNP.

Table S4: GWAS linked traits of 14 instrument SNPs of ADPN.

| SNP | Phenoscanner | dbSNP genes | GWAS catalog traits linked to this gene |
| --- | --- | --- | --- |
| rs2062632 | ADPN, Activated partial thromboplastin time | KNG1 | NA |
| rs17366568 | ADPN | ADIPOQ | ADPN (BMI-adjusted) |
| rs1108842 | ADPN, Impedance of whole body, Activated partial thromboplastin time, Hemoglobin concentration | GNL3 | ADPN, Osteoarthritis |
| rs1597466 | ADPN, Hemoglobin concentration, Hemoglobin concentration | NA | NA |
| rs6810075 | ADPN | MCF2L2P1, [ADIPOQ](https://www.ebi.ac.uk/gwas/genes/ADIPOQ) | ADPN (BMI-adjusted),  Blood protein levels |
| rs7615090 | ADPN | NA | NA |
| rs2980879 | ADPN, Triglycerides, Total cholesterol, HDL cholesterol, Coronary artery disease, LDL cholesterol | TRIB1, LINC02964 | [BMI-adjusted waist circumference](https://www.ebi.ac.uk/gwas/efotraits/EFO_0007789), Triglyceride levels, Total cholesterol levels, [coronary artery disease](https://www.ebi.ac.uk/gwas/efotraits/EFO_0001645) |
| rs7955516 | ADPN | PDE3A-AS1, [LINC02468](https://www.ebi.ac.uk/gwas/genes/LINC02468) | ADPN, HDL cholesterol levels, Systolic blood pressure |
| rs601339* | Mean platelet volume, ADPN,  HDL cholesterol, Hip circumference | KNTC1, HCAR2 | ADPN, [body mass index](https://www.ebi.ac.uk/gwas/efotraits/EFO_0004340), [body fat distribution](https://www.ebi.ac.uk/gwas/efotraits/EFO_0004341), [visceral adipose tissue measurement](https://www.ebi.ac.uk/gwas/efotraits/EFO_0004765) |
| rs7964945 | ADPN | NA | NA |
| rs8042532 | ADPN | NA | NA |
| rs12051272 | ADPN, Plasma high molecular weight adiponectin concentration | CDH13 | Coronary artery disease, ADPN |
| rs2927324 | ADPN, HDL cholesterol | NA | NA |
| rs731839 | ADPN, Triglycerides, HDL cholesterol | NA | NA |

*Indicates instrument SNP with potential pleiotropic and was removed in the final MR analyses

Table S5: GWAS linked traits of 7 instrument SNPs of AD.

| SNP | Trait | dbSNP genes | GWAS catalog traits linked to this gene |
| --- | --- | --- | --- |
| rs1752684 | AD | CR1 | Alzheimer's disease, AD in APOE e4+ carriers |
| rs9381563 | AD, Platelet count, Height,  Red cell distribution width | CD2AP-DT, [B3GNTL1P2](https://www.ebi.ac.uk/gwas/genes/B3GNTL1P2) | AD, Cognitive function, Platelet distribution width, Height |
| rs10808026 | AD | EPHA1 | AD, Blood protein levels, Sex hormone-binding globulin levels |
| rs7982 | AD | CLU | AD, AD in APOE e4+ carriers, Smoking initiation |
| rs10792832 | AD, High light scatter reticulocyte count | LINC02695, RNU6-560P | [Alzheimer disease](https://www.ebi.ac.uk/gwas/efotraits/MONDO_0004975), [body height](https://www.ebi.ac.uk/gwas/efotraits/EFO_0004339), Red cell distribution width, Insomnia |
| rs11218343 | AD | SORL1 | AD, [alcohol consumption measurement](https://www.ebi.ac.uk/gwas/efotraits/EFO_0007878) |
| rs8093731 | AD | DSG2 | AD, Desmoglein-2 levels,  Beta-1,4-galactosyltransferase 6 levels |

Table S6: GWAS linked traits of 15 instrument SNPs of PD.

| SNP | Trait | dbSNP genes | GWAS catalog traits linked to this gene |
| --- | --- | --- | --- |
| rs12934900 | Mean corpuscular hemoglobin, Red blood cell count, Hip circumference | NA | NA |
| rs823106 | Basophil percentage of white cells | NA | NA |
| rs4774417 | NA | NA | NA |
| rs4488803 | NA | NA | NA |
| rs858295 | PD | NA | NA |
| rs6741007 | PD |  |  |
| rs4588066 | NA | NA | NA |
| rs35749011 | PD | [KRTCAP2](https://www.ebi.ac.uk/gwas/genes/KRTCAP2), HMGN2P18 | PD, [glomerular filtration rate](https://www.ebi.ac.uk/gwas/efotraits/EFO_0005208), Prostate cancer |
| rs329647 | Schizophrenia | [SPATA19](https://www.ebi.ac.uk/gwas/genes/SPATA19), IGSF9B | Parkinson's disease, Insomnia, Schizophrenia |
| rs620490 | PD | NA | NA |
| rs7695720 | Serum creatinine |  |  |
| rs4698412 | PD | BST1 | PD, Blood protein levels, Catalase levels |
| rs75646569 | PD |  |  |
| rs58879558 | Red blood cell count, Hemoglobin concentration, Neutrophil count | MAPT | Osteoarthritis, Neuroticism |
| rs10513789 | PD | MCCC1 | PD, |

Table S7: The sample size and power calculations for MR analyses (two-sided α = 0.05).

| **ADPN-AD/PD (binary)** | | | | | | |
| --- | --- | --- | --- | --- | --- | --- |
| Exposure-outcome | Actual N  (outcome GWAS) | Ratio of cases to controls (outcome GWAS) | Observational HR | R^2^ of IVs  (%) | N required for 80% | Power at actual N  (%) |
| ADPN-AD | 54162 | 0.458 | 1.320^[1]^ | 3.66 | 28063 | 97.00 |
| ADPN-PD | 482728 | 0.075 | 1.200^[2]^ | 5.38 | 161449 | 100.00 |
| **AD/PD-ADPN (continuous)** | | | | | | |
| Exposure-outcome | Actual N  (outcome GWAS) | Ratio of cases to controls (outcome GWAS) | Observational β* | R^2^ of IVs  (%) | N required  for 80% | Power at actual N  (%) |
| AD-ADPN | 39883 | / | 0.278^[1]^ | 4.84 | 3788 | 100.00 |
| PD-ADPN | 39883 | / | 0.182^[2]^ | 1.80 | 12712 | 100.00 |

Abbreviations: ADPN, adiponectin; AD, Alzheimer’s disease; PD, Parkinson's disease; GWAS, genome-wide association study; IVs, instrument variables; HR, hazard ratio.

*β equals to ln ^(HR)^.

Table S8: The heterogeneity and sensitivity results of ADPN and NDs relevant traits.

| outcome | No of  SNPs | MR-PRESSO |  | MR Egger intercept | |  | Cochran’s heterogeneity test | | | |
| --- | --- | --- | --- | --- | --- | --- | --- | --- | --- | --- |
|  |  | Global *P* value |  | Intercept | *P* value |  | IVW-Q value | IVW-*P*value | Egger-Q value | Egger-*P* value |
| IL1β | 13 | 0.997 |  | 0.011 | 0.394 |  | 2.964 | 0.998 | 2.183 | 0.999 |
| IL-6 | 13 | 0.854 |  | -0.002 | 0.891 |  | 8.900 | 0.780 | 8.881 | 0.713 |
| TNFα | 13 | 0.441 |  | 0.007 | 0.593 |  | 14.072 | 0.369 | 13.726 | 0.319 |
| IFNγ | 13 | 0.285 |  | -0.006 | 0.691 |  | 16.937 | 0.202 | 16.707 | 0.161 |
| BMI | 13 | 0.150 |  | 0.003 | 0.330 |  | 32.157 | 0.002 | 29.611 | 0.003 |
| IL10 | 13 | 0.797 |  | -0.0136 | 0.447 |  | 24.722 | 0.025 | 23.513 | 0.024 |


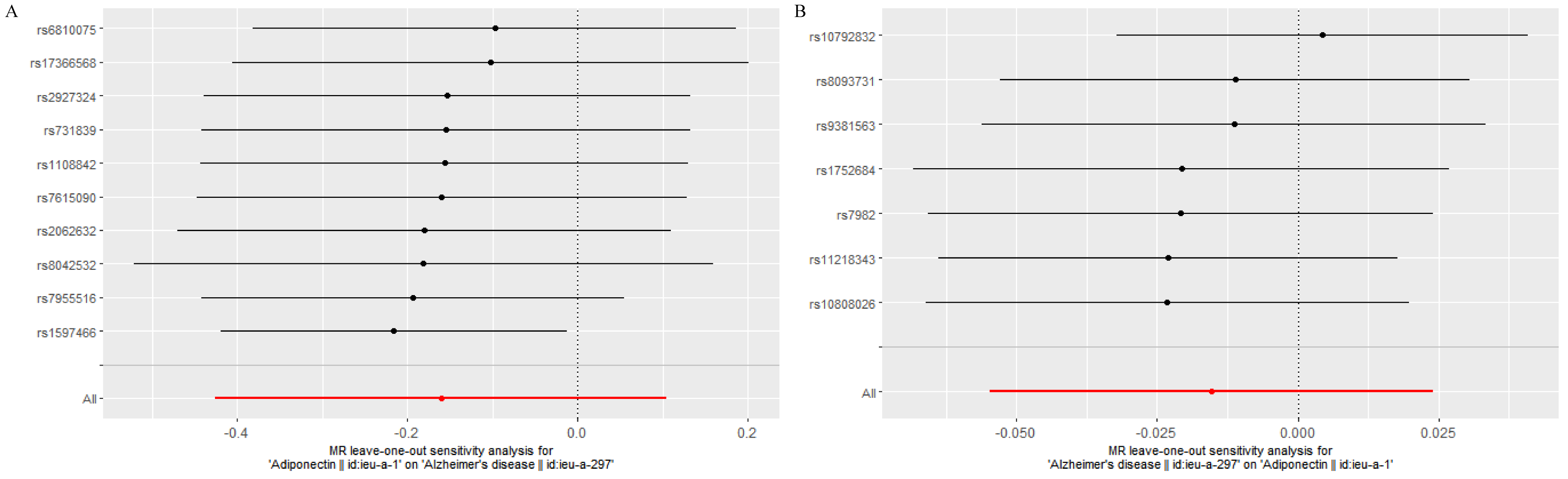


Fig. S1: Leave-one-out plot to assess if a single SNP drives association between ADPN and AD.

1. MR leave-one-out analysis plots for the relationships of ADPN with AD. (B) MR leave-one-out analysis plots for the relationships of AD with ADPN.

Abbreviations: ADPN, adiponectin; AD, Alzheimer’s disease; SNP, single nucleotide polymorphism.


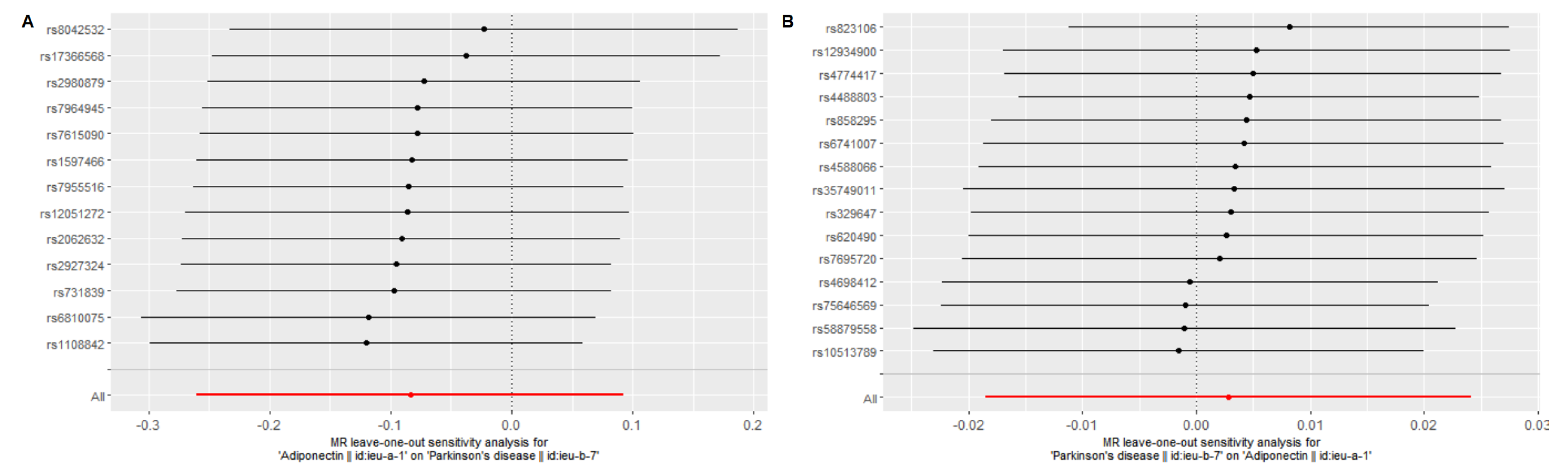


Fig. S2: Leave-one-out plot to assess if a single SNP drives association between ADPN and PD.

1. MR leave-one-out analysis plots for the relationships of ADPN with PD. (B) MR leave-one-out analysis plots for the relationships of PD with ADPN.

Abbreviations: ADPN, adiponectin; PD, Parkinson's disease; SNP, single nucleotide polymorphism.


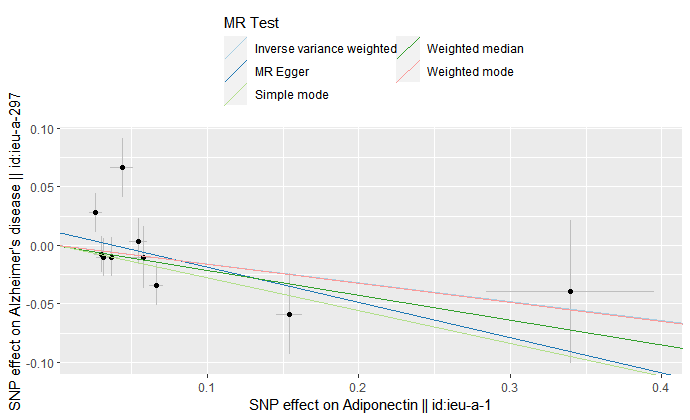


Fig. S3: Scatter plots of causal associations between ADPN with AD.

Abbreviations: ADPN, adiponectin; AD, Alzheimer’s disease

A


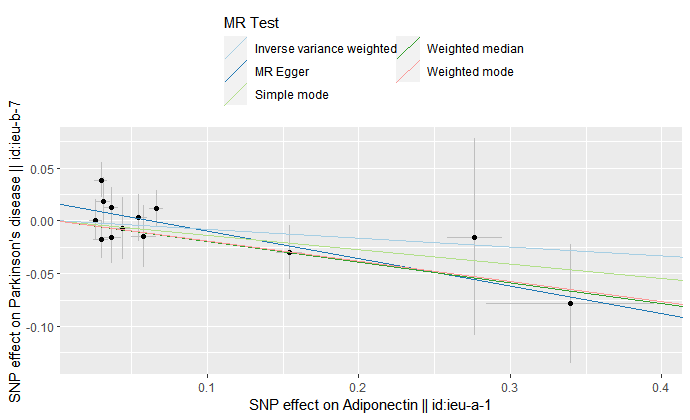


Fig. S4: Scatter plots of causal associations between ADPN and PD.

Abbreviations: ADPN, adiponectin; PD, Parkinson's disease

[Supplementary methods](javascript:;)

1. R^2^ were calculated using the following formula:

(2×EAF×(1-EAF)×beta^2^)

R^2^=

[(2×EAF×(1-EAF)×beta^2^) + (2×EAF×(1-EAF)×N×SE^2^)]

where EAF is the effect allele frequency, beta is the estimated effect on exposure, Ν is the sample size of the GWAS for the SNP-exposure association and SE is the standard error of the estimated effect^[3]^.

1. *F* statistic were calculated using the following formula:

beta^2^

*F=*

SE^2^

where beta is the estimated effect on exposure and SE is the standard error of the estimated effect^[4]^.

Reference

1. Van Himbergen T M, Beiser A S, Ai M, et al. Biomarkers for insulin resistance and inflammation and the risk for all-cause dementia and alzheimer disease: results from the Framingham Heart Study[J]. Arch Neurol, 2012, 69(5): 594-600.
2. Kataoka H, Sugie K. Serum adiponectin levels between patients with Parkinson's disease and those with PSP[J]. Neurol Sci, 2020, 41(5): 1125-1131.
3. Shim H, Chasman DI, Smith JD, Mora S, Ridker PM, Nickerson DA, Krauss RM, Stephens M. A multivariate genome-wide association analysis of 10 LDL subfractions, and their response to statin treatment, in 1868 Caucasians[J]. PLoS One. 2015; 10:e0120758.
4. Pierce BL, Ahsan H, Vanderweele TJ. Power and instrument strength requirements for Mendelian randomization studies using multiple genetic variants[J]. Int J Epidemiol. 2011; 40:740–52.
